# Development of Submergence-Tolerant, Bacterial Blight-Resistant, and High-Yielding Near Isogenic Lines of Popular Variety, ‘Swarna’ Through Marker-Assisted Breeding Approach

**DOI:** 10.3389/fpls.2021.672618

**Published:** 2021-07-27

**Authors:** Shibani Mohapatra, Alok Kumar Panda, Akshaya Kumar Bastia, Arup Kumar Mukherjee, Priyadarshini Sanghamitra, Jitendriya Meher, Shakti Prakash Mohanty, Sharat Kumar Pradhan

**Affiliations:** ^1^ICAR—National Rice Research Institute, Cuttack, India; ^2^School of Applied Sciences, KIIT Deemed to Be University, Bhubaneswar, India; ^3^Department of Botany, North Orissa University, Baripada, India

**Keywords:** bacterial blight resistance, marker-assisted breeding, submergence tolerance, target gene, yield QTL

## Abstract

The rice variety ‘Swarna’ is highly popular in the eastern region of India. The farmers of eastern India cultivate mainly rainfed rice and face the adverse effects of climate change very frequently. Rice production in this region is not stable. Swarna variety is highly susceptible to bacterial blight (BB) disease and flash floods, which cause a heavy reduction in the yield. Transfer of five target genes/QTLs was targeted into the variety, Swarna by adopting marker-assisted backcross breeding approach. Direct markers for *Sub1A, OsSPL14*, and *SCM2* QTLs and closely linked markers for *xa5* and *Xa21* BB resistance genes were screened in the backcross progenies. Swarna-Sub1, CR Dhan 800, and Swarna–Habataki near-isogenic lines (NILs) were used as donor parents in the breeding program. True multiple F_1_ plants were identified for backcrossing, and 796 BC_1_F_1_ seeds were generated. Foreground selection detected all the five target genes in six progenies in BC_1_F_1_ generation. The progeny containing all the target genes and more similar with the recipient parent was backcrossed, and a total of 446 BC_2_F_1_ seeds were produced. Foreground screening detected four BC_2_F_1_ plants carrying the five target genes. A total of 2,145 BC_2_F_2_ seeds were obtained from the best BC_2_F_1_ progeny. Screening of the progenies resulted in one plant with all five desirable genes, three plants with four, and another three progenies carrying three genes in homozygous conditions. The pyramided lines showed higher BB resistance and submergence tolerance than the recipient parent, Swarna. Culm strength of the pyramided lines showed higher breaking force than the recipient parent, Swarna. The pyramided line, SSBY-16-68-69 yielded the highest grain yield of 7.52 t/ha followed by the lines SSBY-16-68-511 (7.34 t/ha) and SSBY-16-68-1633 (7.02 t/ha). The best-pyramided line showed a yield advantage of 18% over the recipient parent and 6.8% over the yield component donor parent. Seven pyramided lines showed higher yield than the recipient parent, while five lines were better yielders than the yield component donor parent. The pyramided line SSBY-16-68-69 produced 365 grains/panicle, while the recipient had 152. The main morphologic and grain quality features of the recipient parent were retained in the pyramided lines.

## Introduction

Rice, the queen of cereals, is the livelihood of millions of the world's inhabitants. Among various food crops, rice is the staple food in many parts of the globe. Rice provides carbohydrates, proteins, dietary fibers, specific oils, vitamins, minerals, and other compounds. The population is increasing at a very rapid rate of around 1.05% per year (Roser et al., 2013). In the coming years, India needs to increase its rice production to fulfill the ever-growing need of the people of the country. The target of additional rice production is about 2–3 million tons of milled rice per year. This increase in production should be achieved from less land, less labor, fewer chemicals, and less water in the coming years which will be a challenging task (Pradhan et al., 2019). Rice can be grown in varied agroecology from high altitude to low altitude. The crop is cultivated in about 161 million ha of global area, and 45% of rice area is under rainfed ecology, which gives low production due to many biotic and abiotic stresses. The rainfed lowland ecology of eastern India, which occupies around 15 million ha, faces submergence stress due to flash floods and suffers from drastic yield loss (Pradhan et al., 2019). Increases in population, affluence, and food preferences have led to global food demand. The worldwide food production target is predicted to be increased by 25–70% by 2050 (Hunter et al., 2017). Resilient rice varieties against biotic and abiotic stresses producing high grain yield are needed for the eastern region to meet the climate change-related effects in this part of the country.

Rice plants face a variety of difficulties in terms of proper growth and survival under submergence stress. Total crop loss is imminent in the cultivation of the submergence susceptible varieties in flood-affected areas like eastern India. In countries like India, Myanmar, and Bangladesh, rice crop yield is mainly affected by instability in rainfall caused by flash floods. Over 16 MHA of the rice cultivated lands of the world are severely affected by complete submergence in low-lying rice areas (Pradhan et al., 2015a). During the wet season, rice grown in India is often submerged during the vegetative stage, sustaining major yield losses. The rice plant is well-adapted to environments with water which is mainly due to aerenchyma tissues. These tissues help in oxygen diffusion by air spaces from root to shoot. This also helps in avoiding the generation of anoxia in the roots. But the rice crop cannot adjust or adapt itself to flash floods. If flooding happens for many days, then it can be fatal for small rice plants. This is a common phenomenon in the low-lying areas of Asia, where monsoon rains are very frequent. The presence of *Sub1A* QTL protects the plant for a 2-week submergence stress tolerance (Pradhan et al., 2019).

Among various biotic stresses, BB disease is a very destructive disease for the rice crop. This disease is caused by *Xanthomonas oryzae* pv. oryzae (*Xoo*). This disease causes yield loss of about 20% to as high as 80%, depending on location, season, and variety (Pradhan et al., 2015b). A total of 45 BB resistance genes have been identified globally to date (Pradhan et al., 2020a). Therefore, high-yielding varieties combined with tolerance to flash floods and BB resistance are highly needed for the rainfed shallow lowland ecology of the eastern region of the country. The future production increase most likely to be achieved under the bad effects of climate change. The current breeding program targets higher production by simultaneous improvement of stress tolerance and yield in rice. The yield of a variety can be increased by incorporating suitable yield genes lacking in a variety. Among the yield component traits, grain number enhancement is controlled by QTLs, *Gn1a* (Ashikari et al., 2005), panicle weight and grain number (Ookawa et al., 2010; Kim et al., 2018; Pradhan et al., 2018), and grain number and culm strength (Ookawa et al., 2010). Transferring these yield component QTLs into a variety lacking these genes will be useful for further raising the yield potential of that variety. Swarna variety is extremely popular in eastern India but is highly susceptible to submergence and BB disease, which can cause a great loss. Therefore, we combined these two characteristics with yield component QTLs to enhance the yield and convert it into biotic and abiotic stress tolerant.

Many susceptible varieties are now successfully incorporated with different genes namely submergence and drought tolerance, BB resistance, and high grain yield (Hattori et al., 2009). Therefore, with the inclusion of suitable genes through gene pyramiding or gene stacking in the popular variety, Swarna will increase rice production in the region. It is difficult to sequentially transfer several resistance genes to a variety with the aid of traditional breeding. The use of linked and direct molecular markers for different traits through gene stacking is an extremely useful strategy to transfer many genes to the recipient parent at the same time. In this investigation, we targeted the Swarna variety as a recipient for incorporation of traits namely submergence tolerance, broad-spectrum BB resistance (*xa5* and *Xa21*), and yield component QTLs (*SCM2* and *OsSPL14*) to make it more resilient and yielding in the eastern region of the country.

## Materials and Methods

### Plant Materials and Hybridization

**Figure 1:**
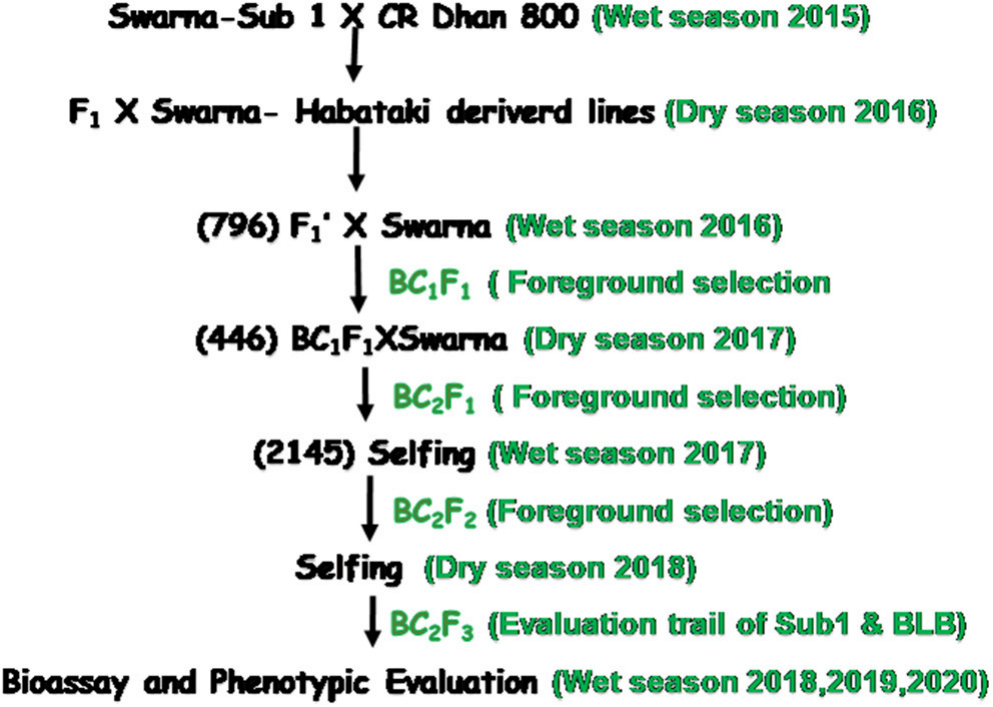
Flowchart for development of submergence tolerant, bacterial blight resistant, and high yielding genotype in the background of popular variety, ‘Swarna’ through the marker-assisted breeding approach.

The donor parent for submergence tolerance used in the breeding program was Swarna Sub-1 which carries Sub-1A QTL. Another donor parent, CR Dhan 800 (Swarna MAS), that contains four BB resistance genes, *viz., Xa21, xa5, xa13, Xa4* in the background of Swarna variety were used in this hybridization program. The third donor parent was a stable NIL of BC_2_F_2_ from Swarna and Habataki backcross used for yield component QTLs, *OsSPL14*, and *SCM2*. During the wet season 2015, F_1_ seeds were generated from Swarna-Sub1 and CR Dhan 800. The generated F_1_ seeds were sown in the shallow pots present in the net house. After 21 days, the seedlings were transferred to the main field for better growth. True hybridity was checked using the *Xa21* marker. A true F_1_ plant was hybridized with the third donor parent, Swarna–Habataki NIL, during the dry season, 2016. This line was selected from the molecular screening results of 72 BC_2_F_2_ progenies based on yield component QTLs screened during the wet season, 2015. The multiple F_1_ plants, i.e., the F_1_ plants obtained from the three-way cross, were genotyped for the presence of *OsSPL14, SCM2, Sub1A, xa5*, and *Xa21*. The multiple F_1_ plants carrying five target genes were backcrossed with the recipient parent, Swarna, during the wet season, 2016, and BC_1_F_1_ seeds were produced. The progenies were screened for the presence of submergence tolerance QTL, bacterial leaf blight disease resistance genes, and yield component QTLs by the foreground selection during the dry season, 2017. The plants produced from the BC_2_F_1_ plant were used for foreground selection, and the positive plants carrying the desirable genes were then carried forward to the BC_2_F_2_ generation during the wet season, 2017. These progenies were screened for the presence of homozygous target genes carrying plants during the wet season, 2017. The plant containing all the five target genes/QTLs was advanced to BC_2_F_3_ generation, and seeds were increased during the dry season, 2018 for evaluation trials. The pyramided lines and the parents were evaluated for different target traits like BB resistance, submergence tolerance, and various yield component traits during wet seasons, 2018, 2019, and 2020 (Figure 1).

### DNA Isolation and Polymerase Chain Reaction (PCR) Amplification

The genomic DNA of backcross progenies was isolated following the CTAB method in each generation (Dellaporta et al., 1983). Amplification of DNA was performed in a Thermal Cycler (Veriti, Applied Biosciences, New York, NY) with the reaction volume of 10 μL containing 50 mM KCl, 1.5 mM Tris HCl (pH 8.75), 1% TritonX-100, 2 mM MgCl_2_, 200 mM each of dCTP, dATP, dGTP, dTTP, 4 pmol of each primer (forward and reverse), 30 ng of genomic DNA, and 1 unit of DNA Taq Polymerase. The reaction mixture was denatured for 4 min at 94°C. In the next step, 1 min denaturation at 94°C was done. After that, 1 min annealing at 55 to 58°C and an extension of 1 min at 72°C were performed. This cycle was repeated 35 times. Then a final extension for 10 min at 72°C was performed. The PCR products of each sample were loaded on 2.5 % agarose gel in 1 × Tris-Borate-ethylenediaminetetraacetic acid buffer to resolute the amplified fragments, and the gel was run at 120 V for around 4 h, and the photograph was taken using a Gel Doc System (SynGene, Bangalore, KA, India). The steps for a polymerase chain reaction, electrophoresis, and gel documentation were performed as per the earlier publications (Pradhan et al., 2016a; Barik et al., 2018; Pawar et al., 2021).

### Markers Analysis

Eight gene-specific and linked markers for the five target genes were used to do the foreground selections (Table 1). These markers were first used for the validation of target genes in the donor parents. The foreground selection was performed up to BC_2_F_2_ generations to ensure the presence of the desired genes. The data analysis, the construction of the similarity matrix from the binary data with Jaccard's coefficients, dendrogram, and principal component analyses were performed following the earlier publications (Barik et al., 2019; Pandit et al., 2020; Pradhan et al., 2020b).

**Table 1:** Molecular markers used for tracking yield component, submergence tolerance QTL, and bacterial blight resistance genes in the backcross progenies.

**Trait/Gene/QTL**	**Chromosome number**	**Marker**	**Primer sequences used for gene detection**		**Expected size (bp)**	**Band type**	**References**
			**Forward(5^**′**^-3^**′**^)**	**Reverse (5^**′**^-3^**′**^)**			
xa5	5	xa5S (Multiplex)	GTCTGGAATTTGCTCGCGTTCG	TGGTAAAGTAGATACCTTATCAAACTGGA	410 bp, 310 bp, 180 bp	STS	Pradhan et al., 2015a,b,c
		xa5SR/R (Multiplex)	AGCTCGCCATTCAAGTTCTTGAG	TGACTTGGTTCTCCAAGGCT			
Xa21	11	pTA248	AGACGCGGAAGGGTGGTTCCCGGA	AGACGCGGTAATCGAAGATGAAA	1,000 bp	STS	Huang et al., 1997
Strong Culm	6	(SCM 2) (F) (SCM 2) (R)	5′ ATTCAGATCAATAGGTTGAGTGT 3′	5′ TGCTATGTATATCCTATCGGTTC 3′	180	Direct	Ookawa et al., 2010
Heavy panicle and plant architecture	8	OsSPL14(F) OsSPL14(R)	5′ CAAGGGTTCCAAGCAGCGTAA 3′	5′ TGCACCTCATCAAGTGAGAC 3′	500	Direct	Miura et al., 2010
Sub1A	9	Sub 1 A203	5′ CTT CTT GCT CAA CGA CAA CG 3′	5′ AGG CTC CAG ATG TCC ATG TC 3′	200	Direct	Xu et al., 2006; Septiningsih et al., 2009; Pradhan et al., 2015a

### Screening for Submergence Tolerance

The experiment was conducted during wet seasons, 2019, 2020, and 2021 for submergence tolerance screening of the 7 BC_2_F_3_, BC_2_F_4_, and BC_2_F_5_ pyramided lines along with parents in the submergence screening tank of ICAR-NRRI Cuttack. Eleven genotypes, including positive and negative checks (both susceptible and resistant checks), were planted in three replications providing 15 plants/row. The genotypes were submerged after 20 days of transplanting in the tank. A water flow using a borewell was maintained to submerge the plants in the shortest time completely. Water depth was checked daily, and water was added to submerge the materials as per the requirement completely. This screening tank was de-submerged after 14 days of submergence. De-submergence was done carefully to avoid lodging of the seedlings due to the fast drain out. Seven days after de-submergence, the regeneration percentage of the plant was counted. Submergence scoring and data analyses were performed as per the earlier publications (Pradhan et al., 2015a, 2019).

### Bioassay Against BB Disease Resistance

Bacterial blight strains inoculation for bioassay of pyramided lines was performed by creating epiphytotic conditions for inoculation of rice seedlings by active strains following the clipping method. First, the scissor was sterilized using 70% ethanol. Then it was dipped into the bacterial suspension, and the upper portion of the test genotype leaf of the plant was cut. Eight BB strains of Odisha state were maintained at NRRI, Cuttack were inoculated to the pyramided and parental lines. At the maximum tillering stage, five leaves from five different plants were inoculated from each entry and replication, and lesion lengths (LL) were recorded after 15 days. The pyramided lines were categorized as resistant (R), moderately resistant (MR), moderately susceptible (MS), and susceptible (S) based on the mean LL. The scoring and classification of pyramided lines were performed as described earlier (Pradhan et al., 2015b, 2019).

### Phenotyping for Culm Strength of the Pyramided and Parental Lines

The 11 pyramided lines and the parental lines were phenotyped for culm strength during the wet seasons, 2019, 2020, and 2021. The internodes from the top third and fourth were cut and bending strength at breaking (BL), and the physical strength of the culm was quantified using a physiological culm strength meter. The inner and outer diameters of the upper and lower end of both the internodes 3 and 4 were measured to calculate the radius of the culm. The culm radius was calculated by subtracting the inner diameter from the outer diameter and dividing it by two. Phenotyping for culm strength was performed as per the earlier publication of Nomura et al. (2019).

### Evaluation of Yield and Other Component Traits of the Pyramided Lines

Thirty days old, pyramided lines carrying *Sub1*, BB resistance genes, and yield component QTLs were transplanted along with the parents in the main field during the wet season, 2019, 2020, and 2021. A plot size of 5.25 m^2^ was allocated for each entry with 35 plants per row and five rows per entry in a randomized complete block design with three replications at 15 × 20 cm spacing. The recorded data were taken from 10 plants of each entry and replication for yield component traits viz., plant height, panicle length, panicle weight, panicle branching, secondary branching, number of seeds per panicle, 1,000-seed weight, while days to 50% flowering and plot yield (t/ha) on the whole plot basis. The observations for phenotypic traits related to yield components and their contributed traits were recorded at crop maturity and post-harvest stages by following the Standard Evaluation System (SES), IRRI (2014). Grain quality parameters such as grain length, grain breadth, milling (%), head rice recovery (%), and kernel length after cooking (mm) of the test entries were estimated following standard procedure. The head rice recovery % of the entries were estimated per the method of Cagampang et al. (1973). Estimation of milling and cooking qualities were performed per the earlier publications (Pradhan et al., 2015c, 2019). All the data analyses were performed using the software, OriginPro (2021).

## Results

### Foreground Selection in Backcross Progenies

**Figure 2:**
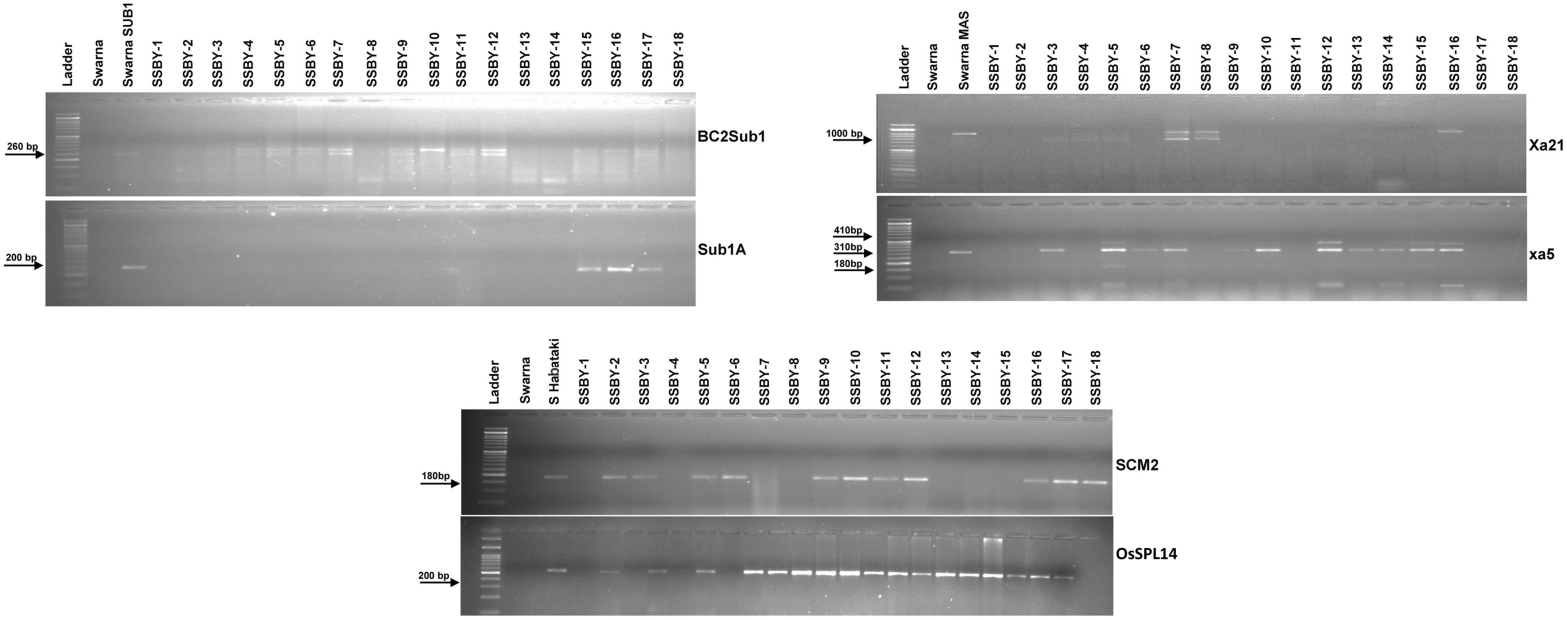
Representative electropherogram of BC_1_F_1_ progenies in the Swarna background for submergence tolerance QTL *Sub1A*, bacterial blight resistance gene *xa5* and *Xa21* and yield component QTLs, *OsSPL14*, and *SCM2*. The designations on the top of the lane indicate the genotype used. L:100 bp DNA ladder.

Direct and tightly linked markers for submergence tolerance, linked markers for BB resistance, and direct markers for the two yield component QTLs were employed to screen the target genes/QTLs in each backcross generation. Single cross F_1_ plants produced from the cross of Swarna-Sub1 with CR Dhan 800 carrying *Sub1A, xa5*, and *Xa21* were validated for the presence of the genes, and the true crossed plant was hybridized with the third donor parent, i.e., Swarna–Habataki NIL for combining the two yield component QTLs, *SCM2*, and *OsSPL14*. A true multiple ${\text{F}}_{1}^{\prime }$ plant was identified and then hybridized with the recipient parent, Swarna, for producing the BC_1_F_1_ seeds. Backcross seeds were then raised, and foreground selection in 796 progenies was performed to select the desirable plants containing all the five target genes and QTLs. Before performing the foreground selection, the molecular markers were checked for parental polymorphism for validation. For analyzing submergence tolerance, the markers Sub1A203 and Sub1BC2 were taken as they showed better resolution than a single marker. Out of 796 plants, 363 Sub1A203 positive plants and 365 plants positive for BC2Sub1 were detected. In this study, it was observed that 343 plants showed amplification for BC2Sub1and Sub1A203. Hence, 343 BC_1_F_1_ plants may contain submergence tolerance QTL. In this present experiment, 341 plants positive for the *xa5* gene and 75 plants positive for the *Xa21* gene individually out of 796 plants were detected. We detected many desirable genes individually, but when it came to the combination of genes with all the three different traits, we found six plants out of 796 which carry all the five genes/QTLs viz., *OsSPL14, SCM2, Sub1A, xa5*, and *Xa21*. Out of those six plants, the plant with more resemblance with Swarna in phenotype was selected (progeny SSBY-16) for the next backcross with the recipient parent, Swarna (Figure 2). A total of 446 BC_2_F_1_seeds were produced for further screening and evaluation. The marker-assisted approach combined with backcross breeding was practiced, and the plants containing all the targeted genes/QTLs were selected in the foreground selection. The genotyping results showed 169 plants positive for *SCM2* and 173 for *OsSPL14* QTLs. The amplification of the Sub1A marker showed the presence of target band size for the submergence tolerance gene by both the markers, Sub1A203 and BC2Sub1. Another RM marker RM 8300, confirmed the presence of the tolerance trait.

**Figure 3:**
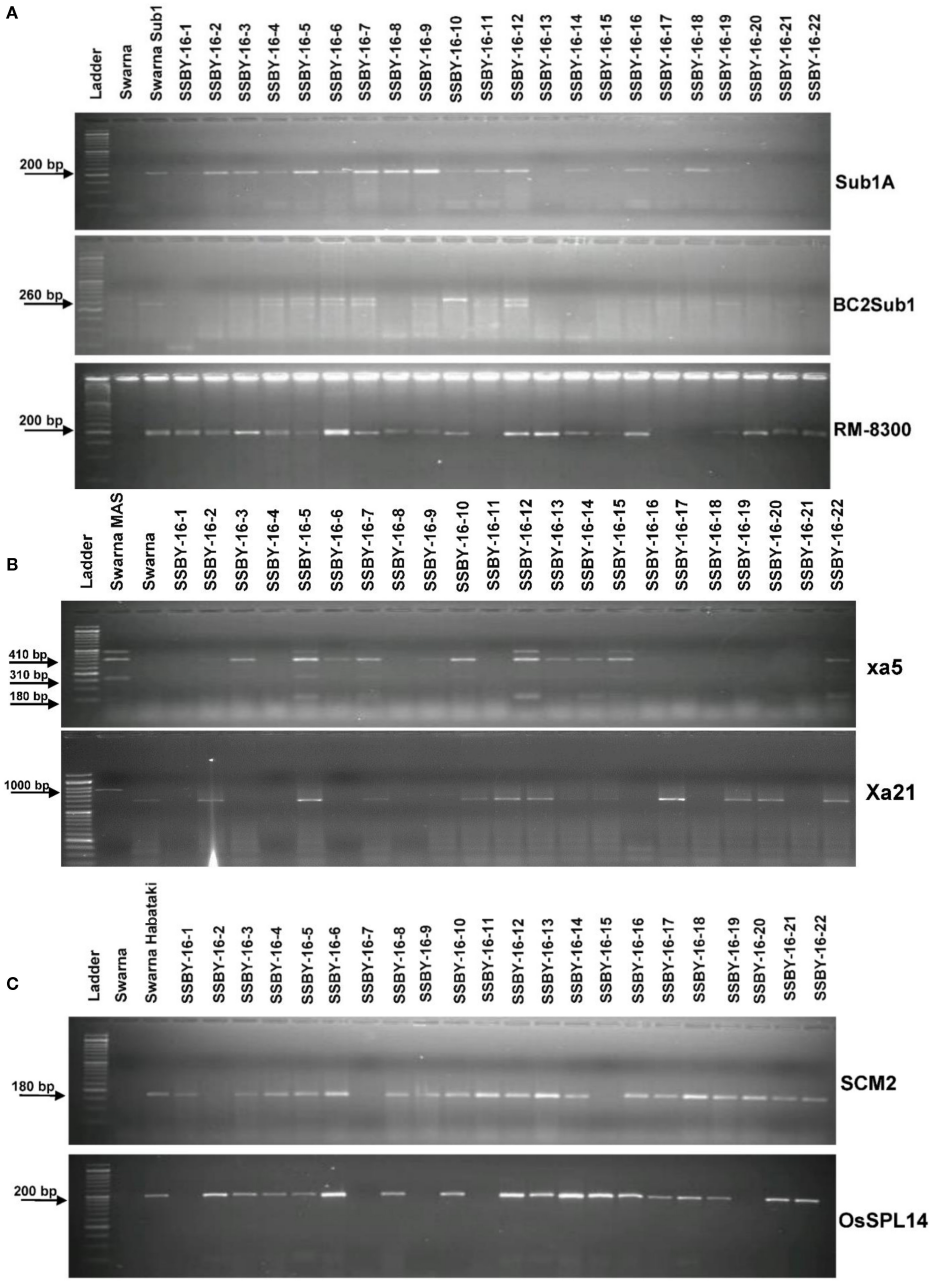
Representative electropherogram of BC_2_F_1_ progenies in the Swarna background for **(A)** submergence tolerance using Sub1A203, BC2Sub1, and RM8300 **(B)** bacterial blight resistance *xa5* and *Xa21* and **(C)** yield component QTLs for *OsSPL14* and *SCM2*. The designations on the top of the lane indicate the genotypes, and the L is 100 bp DNA ladder. The desired base pair of the markers are written on the left.

**Figure 4:**
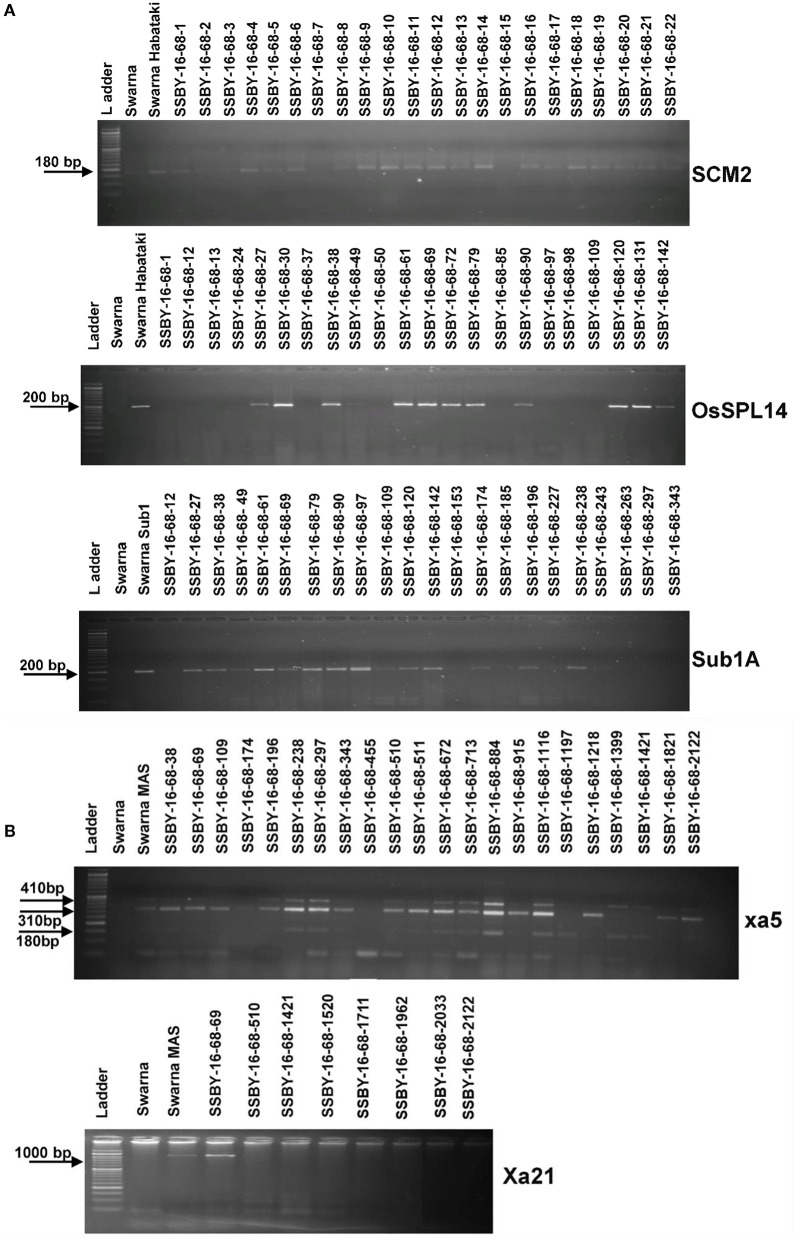
Representative electropherogram of BC_2_F_2_ progenies in the background of Swarna variety for **(A)** yield component QTLs, *OsSPL14*, and *SCM2*, and submergence tolerance **(B)** bacterial blight resistance genes, *xa5* and *Xa21*. The designations on the top of the lane indicate the genotypes used, and the L is 100 bp DNA ladder. The desired base pair of the markers are written on the left.

The analysis detected 159 progenies positive for Sub1A QTL. The two BB resistance genes, namely *xa5* and *Xa21*, indicate durable or broad-spectrum bacterial blight resistance genes in the progenies. In this experiment, 157 and 127 progenies were positive for *xa5* and *Xa21*, respectively. Four plants were found to carry the five target genes/QTLs (Figure 3). The positive plant carrying the five target genes each and similar in appearance with Swarna (SSBY-16-68) was self-pollinated, and 2145 BC2F2 seeds were produced. Foreground selection was performed for the five target genes/QTLs to identify the homozygous plant for the target allele. First, the progenies were screened for yield component QTL, *SCM2*, and we got 513 positive plants. Those 513 plants were used for the next target yield QTL, *OsSPL14*, and we got 122 positive plants. The 122 positive plants were next screened using the *Sub1A* QTL marker, and only 27 plants were positive. The positive progenies carrying three target traits were screened for 2 BB resistance genes sequentially. They were first screened for *xa5* and six positive plants were detected with the four-gene combination. Those six lines were screened for the presence of gene *Xa21*, and we got only one plant positive for all five target genes (*OsSPL14, SCM2, Sub1A, xa5, Xa21*) in a homozygous state. The seeds from the various homozygous positive plants carrying different gene combinations were shelved and used for further evaluation (Figure 4).

### Screening of Pyramided Lines for Submergence Tolerance Under Controlled Conditions

**Figure 5:**
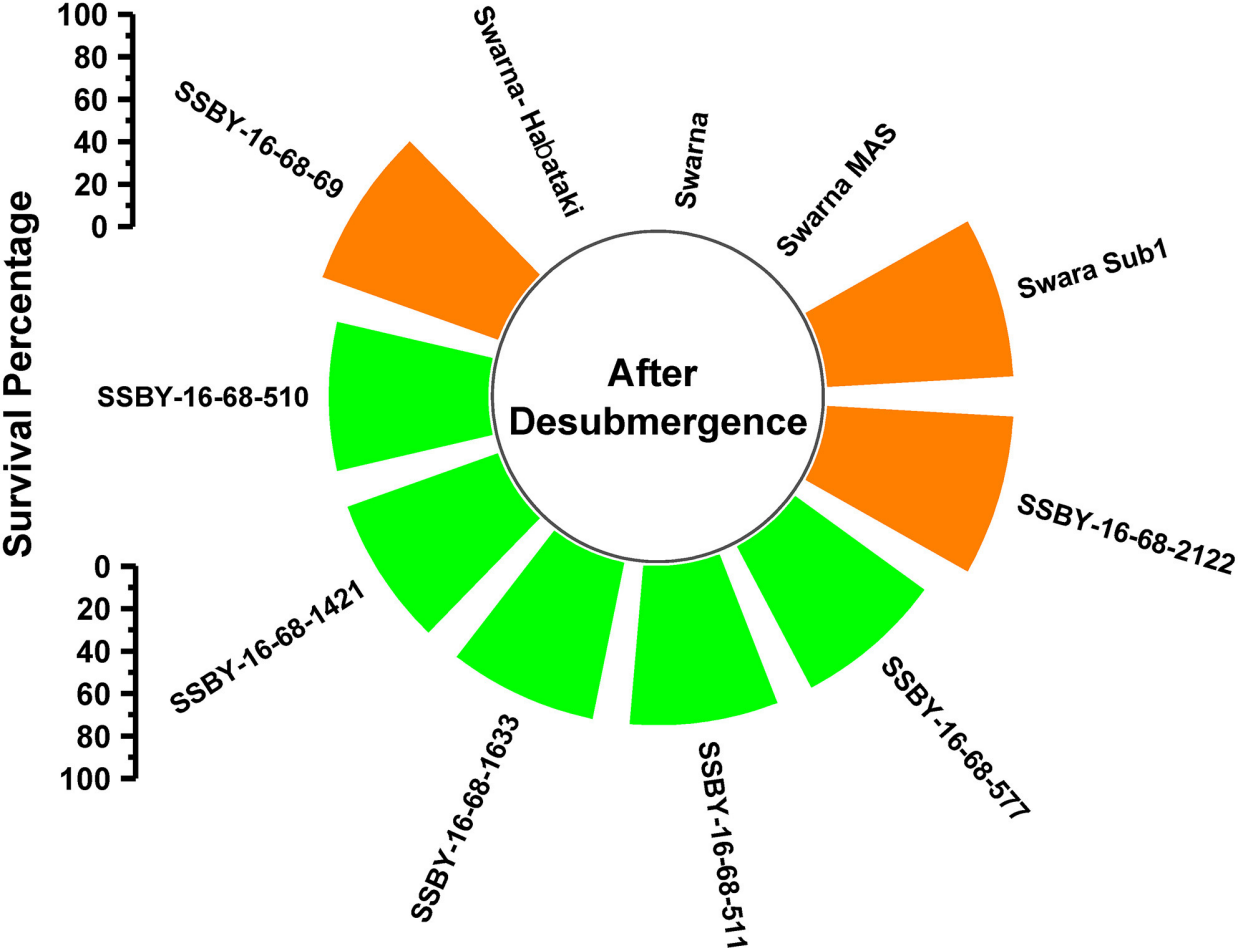
Percentage of plant regenerated under control submergence screening for the *Sub1* QTL carrying lines and its parents after 14 d of submergence stress and 7 days of desubmergence.

A total of 11 genotypes comprising seven BC_2_F_3_ plants carrying five target genes in one plant (*Sub1, xa5, Xa21, SCM2, OsSPL14*), four genes in three plants QTLs (*Sub1, xa5, SCM2, OsSPL14*), three genes in three plants gene/QTLs (*Sub1, SCM2, OsSPL14*) combination plants and four parents were evaluated under controlled conditions in the submergence screening tank. Submergence stress was applied for a total of 14 days on the pyramided and parental lines. The pyramided lines positive for *Sub1* QTL exhibited a 75–87.5% regeneration ability after a week of desubmergence (Figure 5). However, regeneration was not observed in the donor parents Swarna-Sub1, CR Dhan 800, and Swarna–Habataki NIL. Seven pyramided lines showed regeneration ability on par with the donor parent, Swarna-Sub1. The pyramided lines showed better regeneration ability, i.e., 87.5% in SSBY-16-68-69 and SSBY-16-68-2122 (Figure 5).

### Screening of Pyramided Lines for BB Disease Resistance

**Figure 6:**
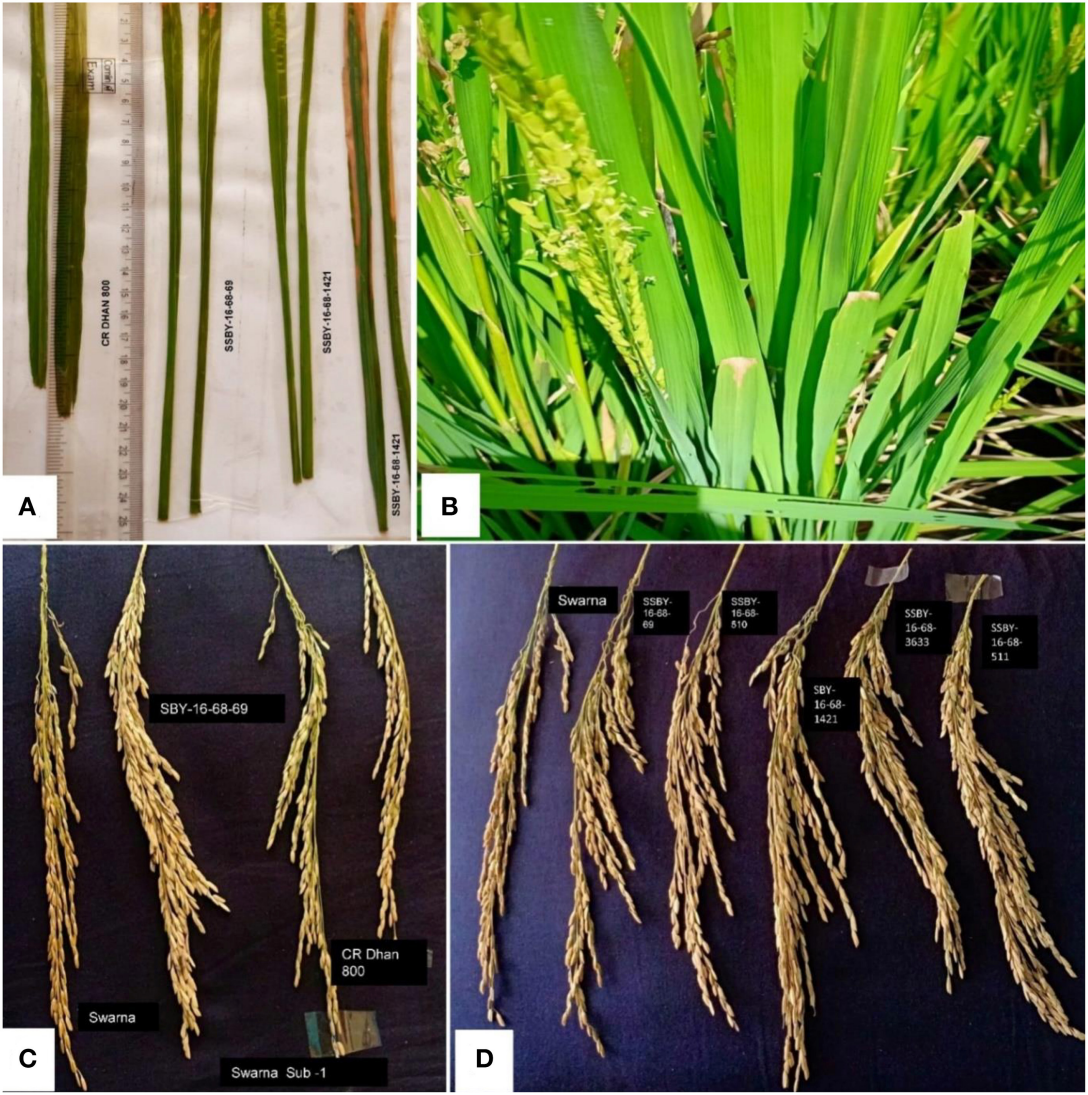
**(A)** Comparative lesion length of donor parent and pyramided lines; **(B)** bacterial blight disease inoculated pyramided plant; and **(C,D)** panicles of donor parents and pyramided lines.

The bioassay was conducted during two seasons of 2019, and 2020 for the resistance reaction of the pyramided lines and parental lines against the eight *Xoo* isolates maintained at NRRI, Cuttack. The results showed that the resistance and the susceptible reaction of the donor (CR Dhan 800) and the recurrent parent (Swarna) are different from each other and depicted in Figures 6A,B. The donor parent showed a smaller range of average lesion lengths (1.4 cm to 1.5 cm), while the variety of Swarna showed a higher lesion length (10.3–12 cm). These findings showed that the pyramid lines were better for bacterial leaf blight tolerance than the recurrent parent, ‘Swarna’ (Table 2). Screening of the BC_2_F_3_ and BC_2_F_4_ pyramided lines against eight *Xoo* isolates exhibited that all the pyramided lines were better than the recipient parent. For BB resistance, different gene combinations containing plants showed a different level of resistance. The lesion length observed varied from 1.3 to 1.5 cm in the pyramided lines carrying *xa5* and *Xa21* resistance gene combinations. Single resistance gene and two-gene combination were tested and showed a different range of lesion length against all BB isolates. Two resistance gene combinations showed better results with the highest resistance, and single gene combination *xa5* showed extremely low resistance. The plant SSBY-16-68-69 showed the highest resistance compared to other plants (Table 2; Figure 6A).

**Table 2:** Bacterial blight (BB) disease score and reaction of pyramided and parental lines against different *Xoo* inoculated strains.

**Sl. No**.	**Pyramided and parental lines**	**Gene combination**	***Xoo*** **strains inoculated**								**Disease reaction**
			**Xa-17**	**Xa-7**	**xa-2**	**xb-7**	**xc-4**	**xd-1**	**xa-1**	**xa-5**	
			**Mean lesion length (cm)**								
1	SSBY-16-68-69	xa5+Xa21	3.8 ± 1.4	3.5 ± 1.23	3.7 ± 1.21	2.9 ± 1.1	3.7 ± 1.45	3.6 ± 1.25	2.4 ± 1.26	2.5 ± 1.17	R
2	SSBY-16-68-510	xa5	9.7 ± 0.72	9.6 ± 0.9	8.2 ± 0.8	9.5 ± 1.3	9.7 ± 0.72	9.6 ± 1.1	8.3 ± 0.8	9.1 ± 0.6	S
3	SSBY-16-68-1421	xa5	9.5 ± 1.9	9.7 ± 0.72	8.3 ± 0.8	9.1 ± 0.6	8.5 ± 1.3	8.7 ± 0.72	8.6 ± 0.9	9.2 ± 0.8	S
4	SSBY-16-68-2122	xa5	8.5 ± 1.3	9.7 ± 0.72	8.6 ± 1.1	8.3 ± 0.8	8.1 ± 0.6	8.7 ± 0.72	8.6 ± 0.9	9.2 ± 0.8	S
5	Swarna MAS	xa5+xa13+Xa21	2.7 ± 0.9	1.9 ± 1.1	2.8 ± 0.3	2.4 ± 0.5	2.7 ± 0.7	2.6 ± 0.7	1.7 ± 1.2	2.2± 0.9	R
6	Swarna-Sub1	_	10.9 ± 1.0	10.5 ± 1.2	10.05 ± 0.6	10.5 ± 0.8	11.5 ± 0.8	12.0 ± 1.3	12.7 ± 1.3	12.3 ± 1.2	S
7	Swarna (recipient)	_	10.9 ± 1.2	10.0 ± 1.3	11.2 ± 1.5	13.7 ± 1.7	13 ± 1.4	12.7 ± 1.4	12.4 ± 1.4	13.7 ± 1.6	S

### Evaluation of Pyramided Lines for Culm Strength

**Figure 7:**
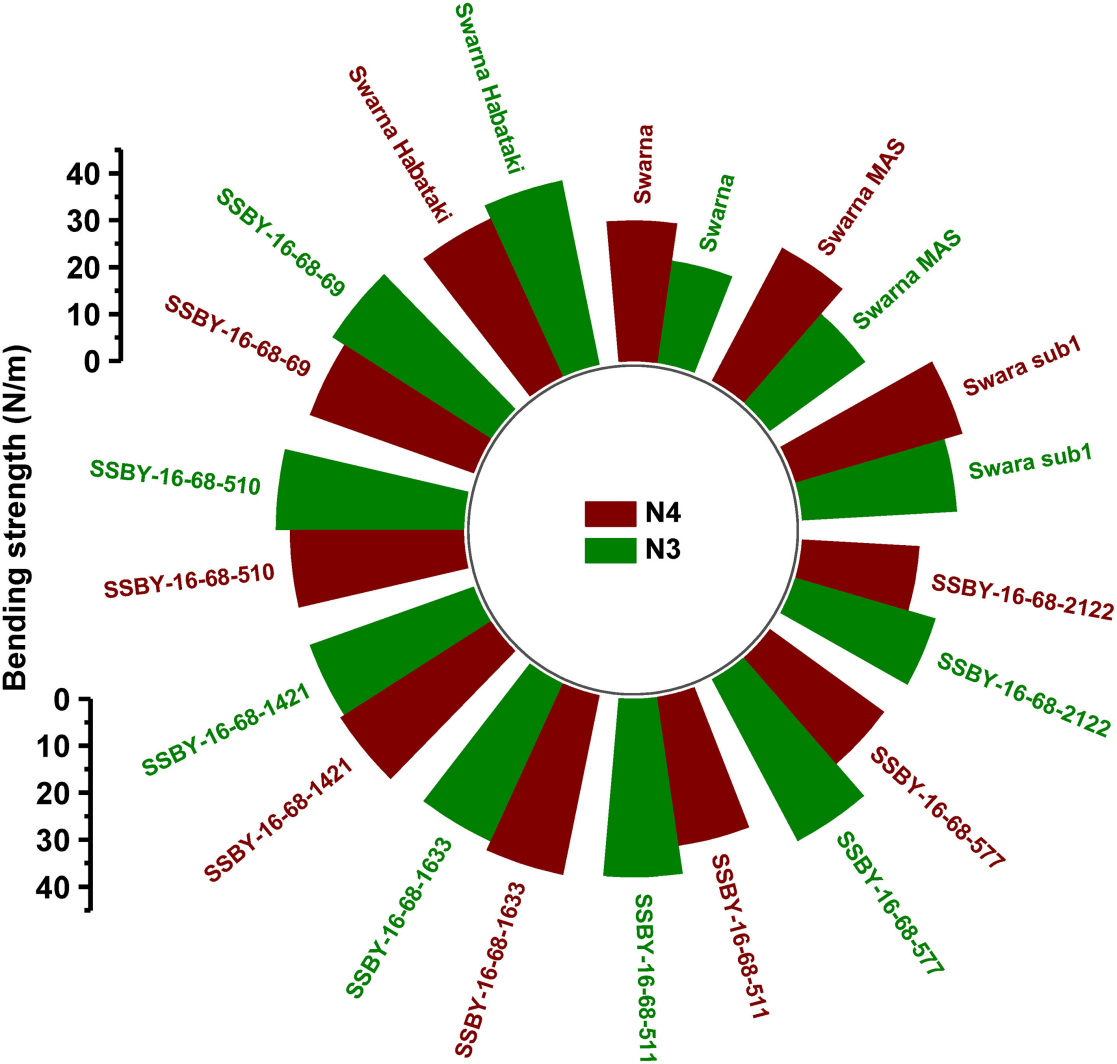
Bending strength of internode N3 and N4 of seven pyramided lines and four parental lines.

**Figure 8:**
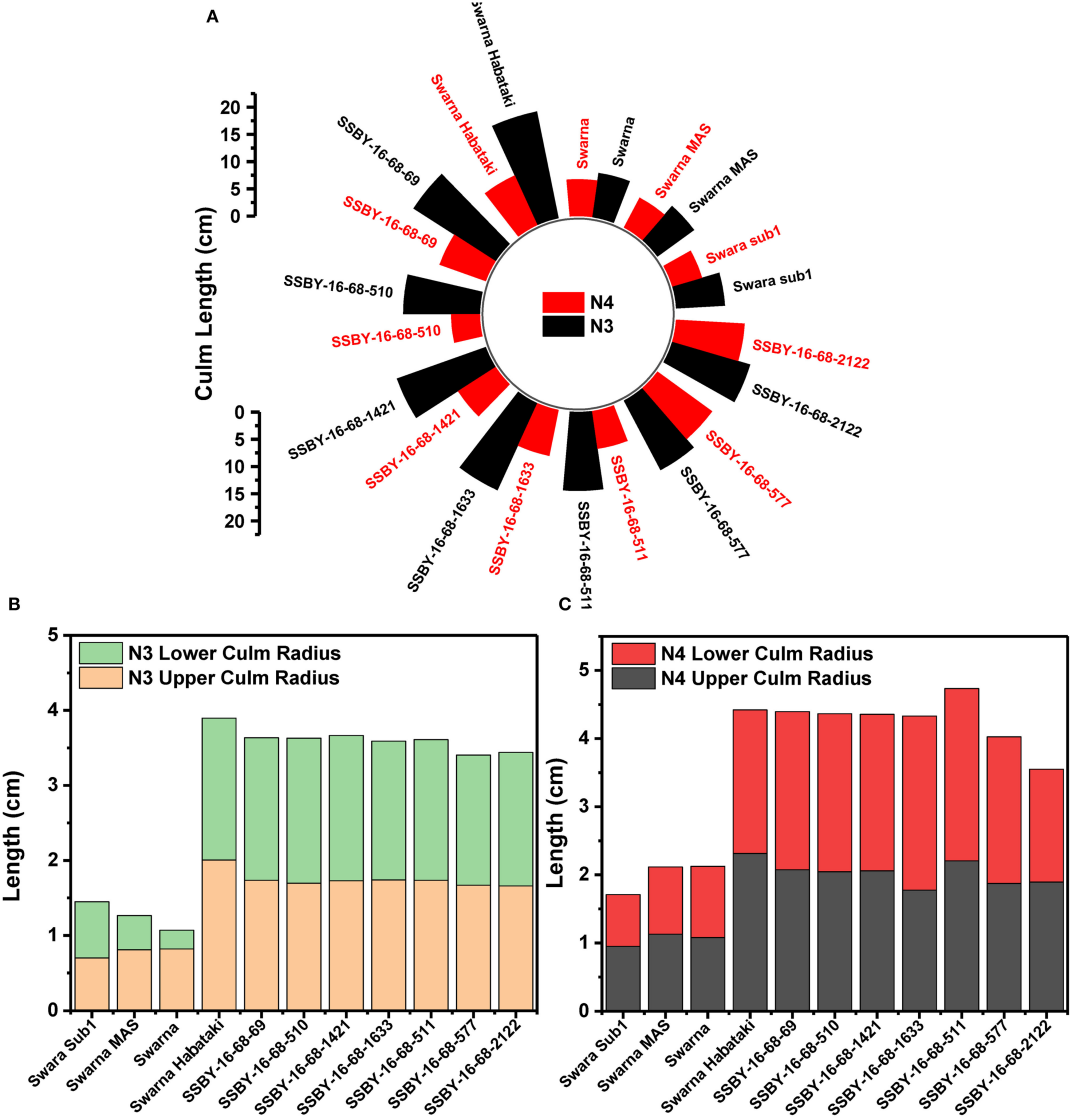
The graphs represent **(A)** culm length and radius of the culm at both upper and lower end of the **(B)** internode N3 and **(C)** internode N4 in the seven pyramided lines and four parental lines.

The graphs for culm strength representing the length of the internode 3 (N3) and internode 4 (N4) for the donor parent Swarna–Habataki derived line and the recipient parent Swarna is presented in Figure 7. We recorded maximum bending strength for Swarna–Habataki derived line, i.e., 40 N/m^2^ and the lowest value of 22 N/m^2^for the recipient parent, Swarna. The pyramided lines exhibited the breaking strength value of 30–40 N/m^2^, which was better than Swarna. The bending strength calculated for SSBY-16-69-3633 showed the highest value of 39 N/m^2^ and the Swarna–Habataki derived line displayed similar bending strength. The highest culm radius in the upper end was 1.7 cm, obtained from the pyramided line SSBY-16-68-69, while Swarna–Habataki derived line and Swarna showed 2.0 and 0.8 cm, respectively. At the lower end, the highest radius was found to be 1.9 cm in the two pyramided lines SSBY-16-68-510 and SSBY-16-68-1421 (Figure 8). The N4 upper end of the parent, Swarna–Habataki derived line, exhibited a culm radius of 2.3 cm, while pyramided line SSBY-16-68-511 showed the highest culm radius of 2.2 cm in the upper end and 2.5 cm in the lower end.

### Evaluation of Pyramided Lines Carrying Genes/QTLs for Submergence Tolerance, BB Resistance, and Yield Component Traits

**Figure 9:**
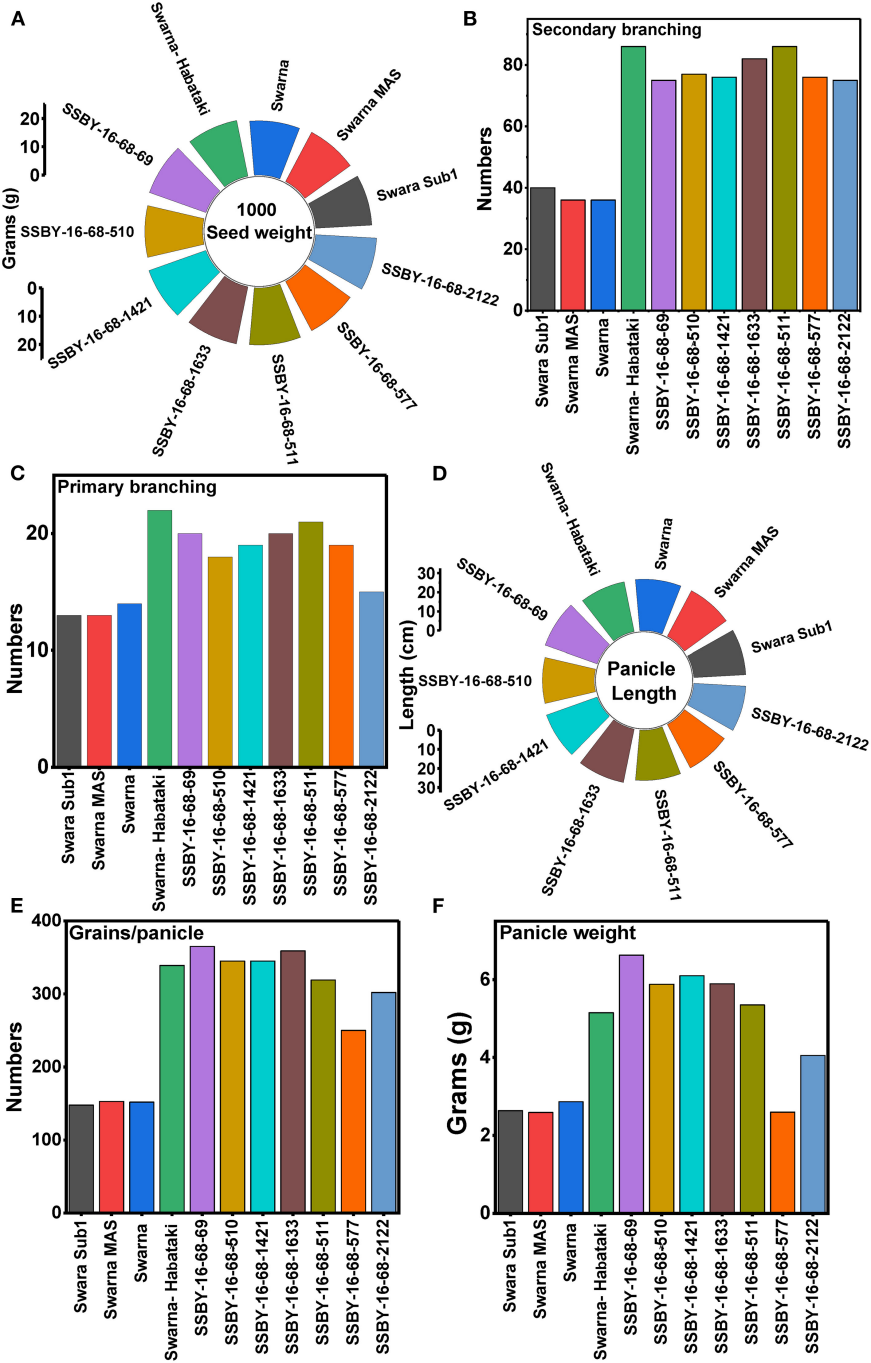
Agro-morphologic and yield parameters of the progenies and parental lines **(A)** 1,000-seed weight; **(B)** secondary branching; **(C)** primary branching; **(D)** panicle length; **(E)** grains/panicle; and **(F)** panicle weight.

In the main field, the pyramid lines containing *Sub1* and BB resistance genes and yield QTLs were transplanted together with the parents and various characteristics, viz., panicle weight, panicle primary branching, secondary branching, grain number per panicle, 1,000-seed weight, panicle length, and plot yield (q/ha) were recorded. Evaluation of pyramided and parental lines indicated that the pyramided line SSBY-16-68-69 showed the highest average panicle weight of 6.63 g followed by 5.89 g in SSBY-16-68-1633 (Table 3; Figures 6C,D). The pyramided line SSBY-16-68-69 also showed the highest grain yield of 7.52 t/ha. The average panicle weight was 2.87 g in the recipient parent, Swarna. The Swarna–Habataki derived donor parent showed an average panicle weight of 5.15 g (Table 3). Five pyramided lines were higher yield than the recipient parent, Swarna, and five were higher than the yield QTLs donor parent, Swarna–Habataki NIL. Field evaluation results indicated that the donor line and the pyramided lines SSBY-16-68-69, SSBY-16-68-511, and SSBY-16-68-1633 exhibited the highest number of primary branches in the panicle (Table 3; Figure 9). The best-pyramided line showed 21 primary branches in the panicle, while the recipient parent had 14 numbers of primary branching (Table 3). The pyramided line SSBY-16-68-511 had 86 secondary branches/panicles, which was on par with the donor parent, Swarna–Habataki derived line. A total of seven pyramided lines with five, four, and three genes/QTLs combination were found to be better than the recipient parent, Swarna, based on the yield-related agro-morphological traits pooled over three seasons (Figure 9).

**Table 3:** Evaluation of pyramided lines for various yield, quality, and related traits in BC_2_F_3_, BC_2_F_4_, and BC_2_F_5_ generations.

**Sl. No**.	**Progenies**	**PH**	**DFF**	**PL**	**SPW**	**NPPB**	**NSBP**	**NGP**	**SF**	**SW**	**GL**	**GB**	**Mill**	**HRR**	**KEAC**	**PY**
1	SSBY-16-68-69	104	117	28.7	6.63	20	75	365	84.5	21.1	5.34	2.32	68.2	64.4	8.06	75.2
2	SSBY-16-68-510	108	117	27	5.88	18	77	345	87.6	20.4	5.35	2.30	67.5	63.5	8.1	69.9
3	SSBY-16-68-1421	105	118	28	6.1	19	76	345	86.7	21.3	5.34	2.34	67.9	68.0	8.07	69.4
4	SSBY-16-68-1633	109	119	28.4	5.89	20	82	359	86.2	20.6	5.35	2.32	68.3	64.4	8.08	70.2
5	SSBY-16-68-511	110	120	26.3	5.35	21	86	319	85.1	20.2	5.33	2.31	68.4	64.3	8.09	73.4
6	SSBY-16-68-577	104	120	25.7	2.6	19	76	250	85.3	19.7	5.38	2.32	67.8	63.1	8.1	63.6
7	SSBY-16-68-2122	106	116	27.1	4.05	15	75	302	87.4	21.5	5.36	2.34	67.1	63.8	8.07	64.5
8	Swarna-Sub1	105	117	27.1	2.64	13	40	148	86.4	19.8	5.33	2.24	68.3	64.3	8.1	56.3
9	CR Dhan 800	106	118	26.9	2.59	13	36	153	86.8	19.7	5.34	2.3	68.9	64.7	8.1	59.2
10	Swarna	108	119	26.8	2.87	14	36	152	86.2	19.2	5.32	2.21	68.5	64.6	8.05	57.1
11	Swarna- Habataki NIL	104	116	26.5	5.15	22	86	339	85.6	20.1	5.38	2.34	68.1	64.2	8.07	68.4
	CV %	3.42	1.34	5.32	9.65	8.4	10.35	10.82	9.12	6.45	0.75	0.14	6.15	7.12	7.426	11.53
	CD_5%_	6.45	7.26	1.52	0.59	1.14	7.24	19.35	7.53	5.92	0.641	0.31	5.21	8.22	1.104	6.25

*PH, plant height (cm); DFF, days to 50% flowering; PL, panicle length (cm); SPW, single panicle weight (g); NPPB, number of primary panicle branches/panicle; NSBP, number of secondary branches/panicle; NGP, number of grains/panicle; SF, spikelet fertility (%); SW, 1,000-seed weight (g); PL, panicle length (cm); GL, grain length (cm); GB, grain breadth (cm); HRR, head rice recovery; KEAC, kernel elongation after cooking (mm) and PY, plot yield (q/ha)*.

**Figure 10:**
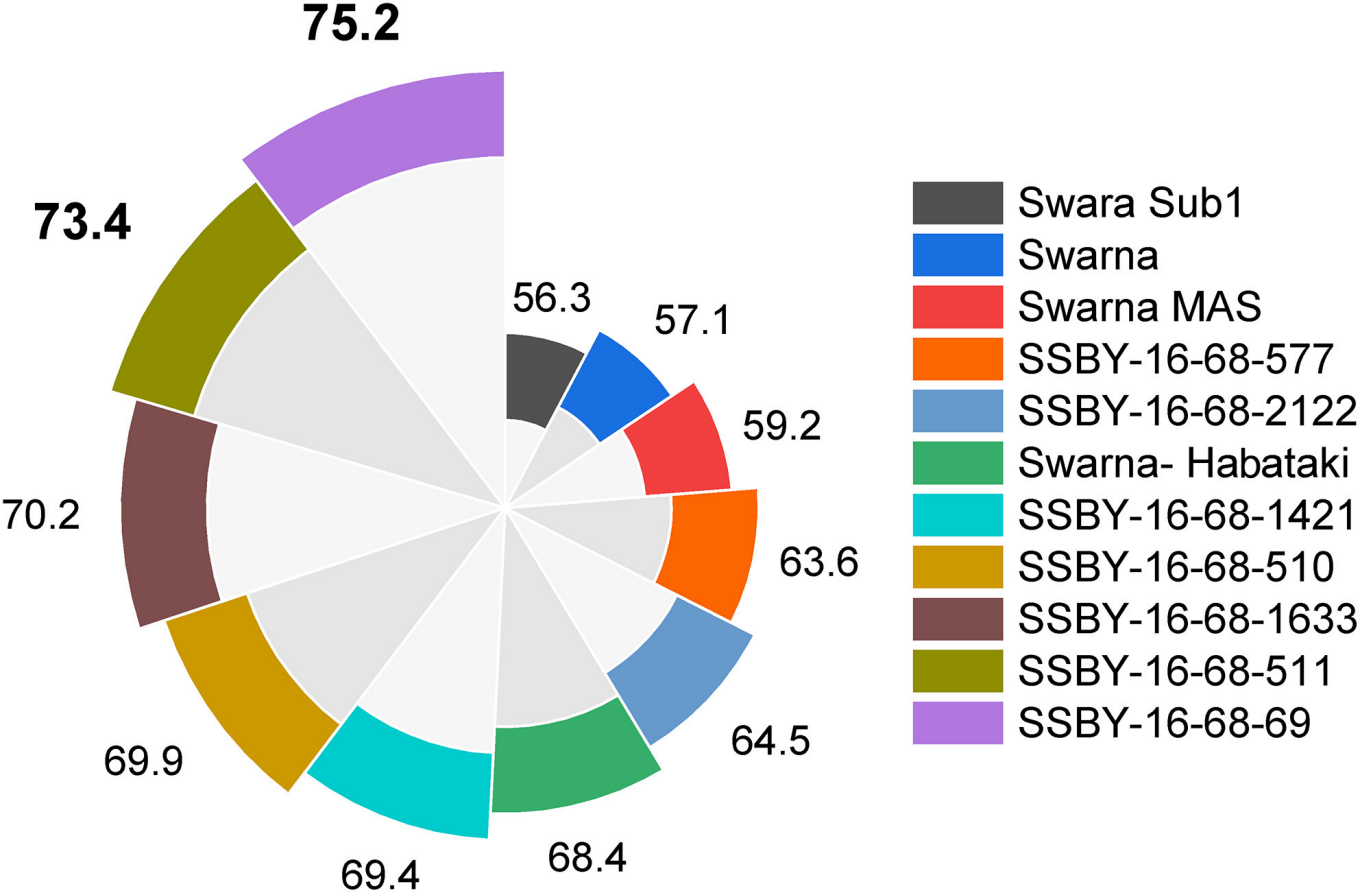
This figure represents yield data in a total of seven progenies with five, four, and three genes/QTLs combination in q/ha.

Evaluation results indicated that the pyramided line SSBY-16-68-69 yielded the highest grain yield of 7.52 t/ha, followed by SSBY-16-68-511 (7.34 t/ha) and SSBY-16-68-1633 (7.02 t/ha), respectively (Table 3). The best-pyramided line showed an advantage of 18% yield over the recipient parent, Swarna, and 6.8% over yield QTLs donor parent, Swarna–Habataki NIL (Table 3). A total of seven and five pyramided lines were found to have a higher yield than the recipient parent, Swarna, and yield QTLs donor parent, Swarna–Habataki derived line, respectively (Figure 10). The pyramided line SSBY-16-68-69 showed 365 average grains/panicle, while the recipient, Swarna, had 152 grains/panicle.

**Figure 11:**
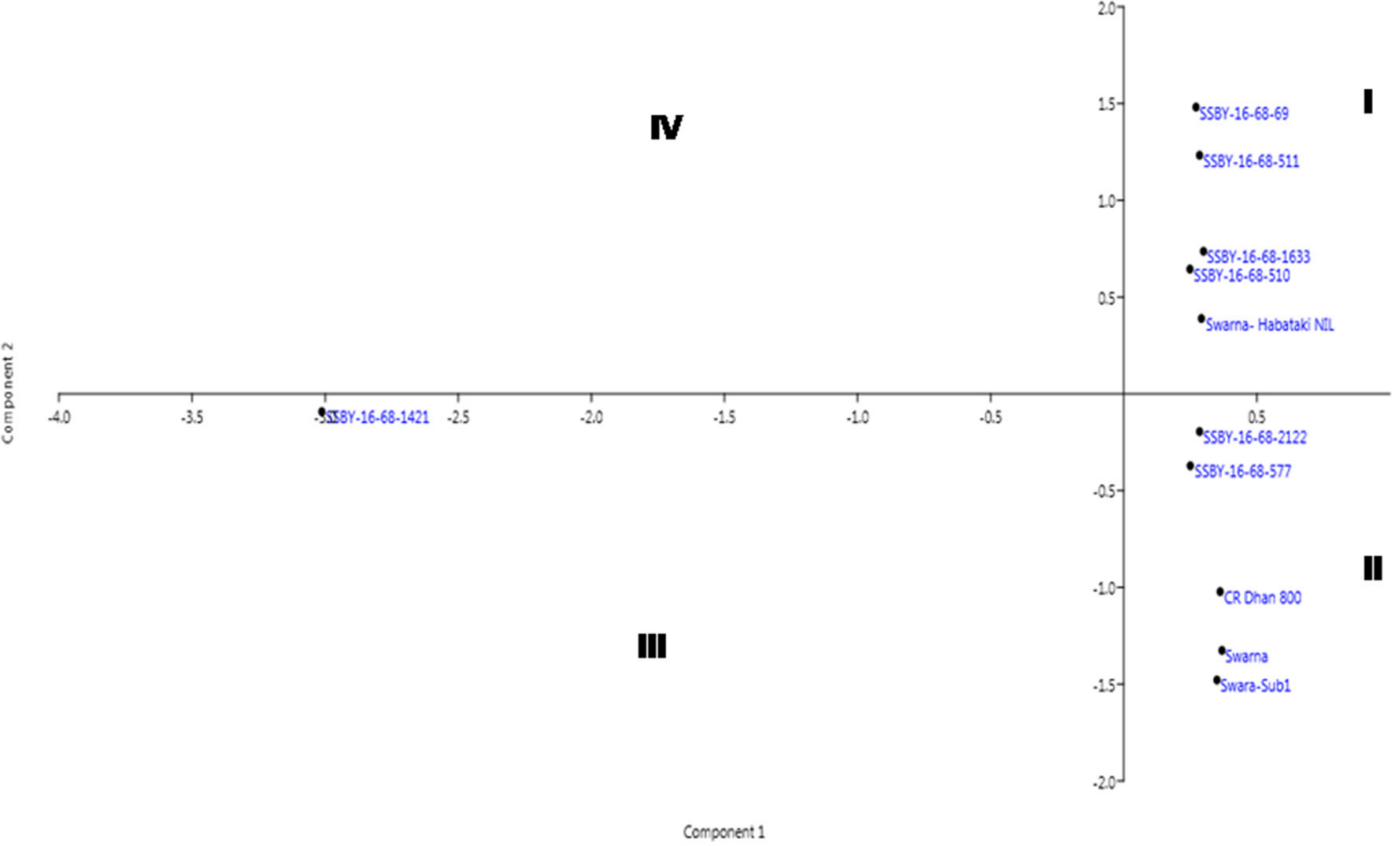
Biplot diagram generated for the traits using the pyramided and parental lines for different traits, viz., SPW, single panicle weight (g); NPPB, number of primary panicle branches/panicle; NSBP, number of secondary branches/panicle; NGP, number of grains/panicle; SW, 1,000-seed weight (g); PL, panicle length (cm); GL, grain length (cm); GB, grain breadth (cm); HRR, head rice recovery, KEAC, kernel elongation after cooking (mm) and PY, plot yield (q/ha) of different Swarna pyramided lines and parental lines.

The pyramided and parental lines were allotted to the four quadrants of the biplot diagram (Figure 11). Four pyramided lines, including the best line, namely SSBY-16-68-511, SSBY-16-68-1633, SSBY-16-68-510, and SSBY-16-68-69, were placed in quadrant I with the yield component parental line Swrana-Habataki NIL. Quadrant II accommodated two pyramided lines SSBY-16-68-212 and SSBY-16-68-577, along with the recipient and two donor parents. In quadrant III, one pyramided line SSBY-16-68-1424 is present. The biplot graph revealed that secondary branches/panicles followed by seeds per panicle and primary branch/panicle exhibited the highest diversity. The pyramided lines present inside the circle need to be tested for release as a cultivar. The pyramided lines present in quadrant I and II were similarly based on the agro-morphologic, and grain quality features such as plant height, days to 50% flowering, spikelet fertility, 1,000-seed weight, panicle length, grain length, grain breadth, head rice recovery, and kernel elongation after cooking (Table 3). The three swarna derived parents were placed very close to each other. In cluster I, the highest yield producing pyramided line was placed, and it is in a different quadrant from recipient parent ‘Swarna’, which is lower in yield and other related component traits.

## Discussion

The rice variety ‘Swarna’ is highly popular in the eastern region of the country. It matures in 145 days and fits the late maturing rice group. During normal weather years, the variety produces exceptionally good yields across the region. But, under unfavorable weather occurrences, the yield from this variety reduces drastically. The occurrences of drought and flood during the cropping season are frequent in the eastern region of India. The farmers of eastern India mostly cultivate rice under rainfed conditions and face the adverse effects of climate change which are very frequent. Thus, rice production in the region is not stable. Again, rice consumers are increasing day by day. Thus, the incorporation of *Sub1* QTL with yield component QTLs is important to enhance the submergence tolerance and grain yield in this popular variety. Bacterial blight disease is also a biotic constraint for higher rice production. Therefore, resistance genes need to be pyramided in the popular varieties for maintaining the productivity of the variety.

Conventional backcross breeding for pyramiding of five target genes in ‘Swarna’ background would take more than 10 years as there are three donor parents, and all are late duration in nature. But, through marker-assisted backcrossing, we were able to develop the variety much earlier with almost half-time duration than the conventional backcrossing. Hence, we have saved a lot of time in this process. Also, we have eliminated the use of chemicals for control of the BB pathogens. The chemicals used in eliminating the pathogens are highly harmful and cause pollution to the environment. Two resistance genes combinations of *Xa21*+*xa5* also showed broad-spectrum resistance to BB pathogen attack (Pradhan et al., 2016b). This approach for host plant resistance is environmentally friendly. Many earlier studies have proposed host plant resistance approach for generating different disease resistance in rice plants (Huang et al., 1997; Rajpurohit et al., 2011; Dokku et al., 2013; Suh et al., 2013; Pradhan et al., 2015b; Pandit et al., 2020). So far, many high yielding BB susceptible rice cultivars, namely PR 106 (Singh et al., 2012); Samba Mahsuri (Arunakumari et al., 2016); Jalmagna (Pradhan et al., 2015b); Swarna with submergence tolerance and bacterial blight resistance (Pradhan et al., 2019), etc. have been improved through marker-assisted selection. But, in this case, we have stacked three different traits into the background of the ‘Swarna’ variety.

The risk of false selection in the recommendation between the molecular marker and the gene/QTLs of interest is reduced by marker-assisted backcross breeding using functional markers. In this study, we were successful in combining submergence tolerance QTL (*Sub1A*), two BB resistance genes (*xa5* and *Xa21*), and two yield component QTLs (*OsSPL14* and *SCM2*) in the late-maturing popular variety, Swarna. *Sub1A* can provide submergence tolerance and transferred using two supporting markers BC2Sub1 and RM-8300. In addition, it also provides tolerance in the form of regeneration or increased survival percentage from the flash flood, which ranges from 75 to 87%. Combining *xa5* and *Xa21* showed durable and broad-spectrum resistance against bacterial leaf blight disease compared with single resistance gene deployment. Two yield QTLs *SCM2* and *OsSPL14* enhanced the yield potential of Swarna. In the vegetative stage, *OsSPL14* regulates shoot branching and is impaired by microRNA excision (Ookawa et al., 2010). We have also shown the feasibility of using the *OsSLP14* WFP allele to increase rice crop yield (Luo et al., 2012). After the incorporation of this QTL, we observed remarkable yield enhancement. To give support to the plant to bear the increased weight of the grains, we incorporated *SCM2* QTL. Recent experimental field hit by typhoon harboring Koshihikari and NIL-SCM2 in 2009. The strong wind due to the typhoon led to the lodging of the Koshihikari did not affect the NIL-SCM2 plants and they remained stationary despite serious lodging due to strong wind (Nomura et al., 2019). The plant we used as the donor parent was Swarna–Habataki NIL carrying both *SCM2* and *OsSPL14*, which produced around 300 grains/panicle, yet its plant architecture supported the heavy panicles. The thickness and the diameter of the culm of Habataki are wider and thicker at the center of the internodes as compared to our recurrent parent Swarna. This validates that our pyramided lines have similar features to our donor parent Swarna–Habataki NIL and are much superior to our recurrent parent in terms of culm strength. The pyramided lines were almost similar in phenotype with the popular recipient variety and hence the pyramided lines are expected to spread quickly and become more popular than the recipient variety. The recovery of the recipient parent genome was accelerated much faster than conventional breeding (Pradhan et al., 2019).

Rice breeding for precise transfer of various traits using molecular markers into popular rice varieties has been reported in many earlier studies (Huang et al., 1997; Rajpurohit et al., 2011; Bhasin et al., 2012; Dokku et al., 2013; Suh et al., 2013; Das and Rao, 2015; Pradhan et al., 2016b). However, in this present molecular breeding program, the developed pyramided lines were observed to carry five target genes from three donor parents that showed tolerance to submergence up to 14 d, resistance to bacterial blight disease, and higher yield than the recipient parent. In an earlier study of Pradhan et al. (2019) they reported developing pyramided lines in the background of the Swarna variety carrying *Sub1, Xa21, xa5*, and *Xa4* but no yield component QTLs. Other research studies (Das and Rao, 2015) reported gene stacking in the Tapaswini and Lalat varieties for submergence tolerance and BB resistance. However, these recipient varieties are early to mid-early type maturing varieties and are not suitable for the late maturity group for eastern India. But, this breeding program for gene stacking was carried out in a very highly popular variety of late-maturing groups and suitable for the eastern Indian situation. In many other studies, marker-assisted breeding was employed for pyramiding target resistance genes for insect pests and diseases in rice (Huang et al., 1997; Sundaram et al., 2007; Rajpurohit et al., 2011; Bhasin et al., 2012; Pradhan et al., 2015b, 2016b; Sharma et al., 2019).

The 7 elite pyramided lines carrying genes for submergence tolerance, high yield potential and BB resistance were SSBY-16-68-69, SSBY-16-68-510, SSBY-16-68-1421, SSBY-16-68-1633, SSBY-16-68-511, SSBY-16-68-577, SSBY-16-68-2122 identified from BC_2_F_2_ generation. Out of those 7 BC_2_F_2_ progenies, the pyramided lines, viz., SSBY-16-68-69 and SSBY-16-68-2622 showed better regeneration ability than the other five pyramided lines. The donor parent showed smaller lesion lengths (0.4–0.5 cm) than Swarna, and the recurrent parent had a higher lesion length (10.3–12 cm) while the pyramided line SSBY-16-68-69 showed the best tolerance with two BB resistance genes. In addition, these pyramided lines were similar in the main agro-morphologic and grain quality features, namely plant height, days to 50% flowering, spikelet fertility, 1,000-seed weight, panicle length, grain length, grain breadth, head rice recovery, and kernel elongation after cooking of the popular variety, Swarna (Table 3). Hence, the pyramided lines are better than the recurrent parent, ‘Swarna’, for bacterial leaf blight tolerance. Our results indicated that the highest grain yield was recorded from the pyramided line SSBY-16-68-69 (7.52 t/ha) followed by SSBY-16-68-511 (7.34 t/ha). Gene pyramiding results of Jena. et al. showed a yield enhancement of 28.4–83.5% by adding *OsSPL14* QTL to it while we got an 18% yield advantage in our best-pyramided line over the recipient parent, Swarna, and 6.8% over yield QTLs donor parent.

## Conclusions

Deployment of a single resistance gene in breeding a variety is risky as resistance knockdown by the pathogen is high. The new, improved pyramided lines showed better resistance to BB pathogens in the background of the highly popular variety Swarna. These pyramided lines are similar to the recipient parent for main agro-morphologic and grain quality features suitable for eastern India. The pyramided lines will give a solution for flash floods and BB endemic areas of eastern Indian regions. The yield enhancement by adding the yield QTLs, *OsSPL14*, and *SCM2* will further increase rice yield, and the gap between the demand and supply of rice will reduce in the future. Wind speed is high in eastern India. Pyramided lines with *SCM2* will give strength to the high-yielding lines. These pyramided lines are expected to impact submergence tolerance, bacterial blight resistance, and higher yield potential than ‘Swarna’ and will emerge as a better option for the farmers.

## Data Availability Statement

The original contributions presented in the study are included in the article/supplementary material, further inquiries can be directed to the corresponding author.

## Author Contributions

SP contributed to the planning, designing, screening, evaluation, and writing of the article. SM and AP performed the genotyping and data analysis. SPM recorded the phenotypic data. AM performed the disease scoring. AB supervised the work. PS Performed the interpretation of data and manuscript preparation. JM and SPM performed the phenotyping for yield component traits of the experimental material. All the authors have read and approved the final manuscript.

## Conflict of Interest

The authors declare that the research was conducted in the absence of any commercial or financial relationships that could be construed as a potential conflict of interest.

## Publisher's Note

All claims expressed in this article are solely those of the authors and do not necessarily represent those of their affiliated organizations, or those of the publisher, the editors and the reviewers. Any product that may be evaluated in this article, or claim that may be made by its manufacturer, is not guaranteed or endorsed by the publisher.
